# Gasotransmitters and Stomatal Closure: Is There Redundancy, Concerted Action, or Both?

**DOI:** 10.3389/fpls.2016.00277

**Published:** 2016-03-15

**Authors:** Denise Scuffi, Lorenzo Lamattina, Carlos García-Mata

**Affiliations:** Laboratorio de Fisiologia Molecular e Integrativa, Instituto de Investigaciones Biologicas-CONICET, Universidad Nacional de Mar del PlataMar del Plata, Argentina

**Keywords:** gasotransmitter, nitric oxide, hydrogen sulfide, guard cell signaling

The epidermis of the aerial part of land plants is pierced by pores through which plants perform gas exchange with environment. The guard cells (GCs), the specialized cells that surround the pore, have the capacity to sense diverse environmental and endogenous stimuli and integrate them into a single output which is the regulation of the stomatal pore width. The stomatal pore size is modulated by changes of the guard cell volume, driven by variations in the osmotic potential of the GCs.

The stress hormone abscisic acid (ABA), the master regulator of stomatal movement, induces stomatal closure by the inhibition of H^+^-ATPases and activation of rapid and slow anion channels, producing the depolarization of the plasma membrane (PM) in GCs, and by an increase in the cytosolic Ca^2+^ concentratrion [Ca^2+^]_cyt_. While the rise of the [Ca^2+^]_cyt_ blocks the influx of K^+^ by the inactivation of the inward rectifying K^+^ channels (K${}_{\text{in}}^{+}$), the depolarization of the PM, in turn promotes K^+^ efflux driven by outward rectifying K^+^ channels (K${}_{\text{out}}^{+}$; Blatt, 2000). This process is closely regulated by a complex signaling network that involves the participation of numerous ubiquitous signaling components like ROS, protein kinases, phospholipases, and protein phosphatases (Kim et al., 2010; Song et al., 2014); and by other signaling components that are emerging as active players in this signaling network, such is the case of gasotransmitters (García-Mata and Lamattina, 2013).

A gasotransmitter is a small gas molecule that: (i) can freely permeates biological membranes; (ii) it is endogenously generated by specific enzymes; (iii) it has specific functions at physiologically relevant concentrations; (iv) it functions can be mimicked by exogenous application of a donor; and (v) it has specific cellular and molecular targets (Wang, 2002). The group of gasotransmitters is, so far, composed by Nitric Oxide (NO), Carbon Monoxide (CO), and Hydrogen Sulfide (H_2_S) and the three of them have been reported to participate in the promotion of stomatal closure (García-Mata and Lamattina, 2013), however, the biology of CO in this physiological process is less known than that of NO and H_2_S. Therefore, this opinion will be focused mainly on the action and interaction of NO and H_2_S. The two of them are accepted as active players in the regulation of stomatal movement, however there are still obscure points and some of them will be discussed in this opinion article: (i) their specific molecular targets; (ii) the molecular mechanisms underpinning their action; (iii) the interplay between them during the stomatal closure induction; and (iv) the crossed-regulation of their metabolism.

All the three gasotransmitters are synthesized during the promotion of stomatal closure. CO is synthesized via the activity of heme oxygenase (Shekhawat and Verma, 2010). In *Vicia faba* CO induces stomatal closure in a dose-dependent manner and acts upstream of the production of NO during ABA-dependent stomatal closure (Cao et al., 2007; She and Song, 2008). It has been reported that the hormones ABA and ethylene (Eth) require the production of NO for the regulation of stomatal movement (García-Mata and Lamattina, 2002; Neill et al., 2002; He et al., 2011; Song et al., 2011). NO can be synthesized either from NO_2_ by two genes, *NIA1* and *NIA2*, that code for a nitrate reductase (NR), or from L-arginine in a reaction catalyzed by an enzyme with nitric oxide synthase (NOS)-like activity, even though the involved enzyme named AtNOA1 possesses GTPase activity (Moreau et al., 2008). However, it was reported that the Arabidopsis triple mutant *nia1/nia2/atnoa1*, which produces very low levels of NO, is hypersensitive to ABA (Lozano-Juste and León, 2010), suggesting that NO could have a dual role in ABA-dependent responses. Moreover, it has been found that increased levels of NO are dependent on the NADPHox-dependent production of H_2_O_2_ (Bright et al., 2006). NO levels can also be modified by an Alternative Oxidase (AOX). Tobacco plants lacking AOX show high NO levels that impacts in stomatal function (Cvetkovska et al., 2014). Recently García et al. (2010) have shown that Arabidopsis mutant plants in β-Cyanoalanine synthase (Cys-C1), a mitochondrial enzymatic source of H_2_S, show higher *AOX1a* transcript levels than wild type, however exogenous application of H_2_S to rice cell culture induced AOX expression (Xiao et al., 2010). Further physiological studies are needed to clarify this interaction.

H_2_S is produced during the passage of L-cysteine to pyruvate and ammonia in a reaction catalyzed by L-cysteine desulfhydrase (DES1; Alvarez et al., 2010). In Arabidopsis there are three different genes involved in this reaction: the *DES1* gene (Alvarez et al., 2010; Scuffi et al., 2014), the *At-LCDES* gene (Jin et al., 2011), and *L-CDes* gene (Hou et al., 2013). Recently, it has been shown that the expression of these genes is upregulated in response to ABA, Eth, JA, and SA, all hormones that modulate stomatal movement (Hou et al., 2013). Even if sequence analysis has shown that the promoter region of the *DES1* gene contains ABA-responsive elements (Scuffi et al., 2014), further work is needed in order to have a better understanding about the mechanism by which these hormones induce the expression of those genes. Although, DES1 was reported to mediate ABA-dependent stomatal closure (Scuffi et al., 2014), it was recently reported that H_2_S regulates the activity of K${}_{\text{in}}^{+}$ channel mostly in an ABA- and Ca^2+^-independent manner, suggesting the existence of ABA-regulated signaling pathways that can be, alternatively, activated in response to other stimuli (Papanatsiou et al., 2015).

## NO physiology in guard cells

More than a decade of work on the participation of NO on the regulation of stomatal movement resulted in a more or less bounded idea of its mechanism of action, in particular in those events triggered by ABA. As stated above, ABA-dependent ROS production induces NO synthesis via NR/NOS-like activities. NO regulates the activity of K${}_{\text{in}}^{+}$ either via the Ca^2+^ release from intracellular Ca^2+^ stores, or through the production of phospholipase D (PLD)-dependent inositol phosphates. Another molecular target of NO is the soluble guanylate ciclase (sGC) that generates cyclic guanosine monophosphate (cGMP) which is converted to 8-nitroguanosine 3′,5′-cyclic monosphosphate (8nitro-cGMP) by NO, modulating the [Ca^2+^]_cyt_ (Joudoi et al., 2013). Recently, it has been proposed that NO may break the ABA signaling in guard cells. On the one hand NO mediates ABA-dependent stomatal closure via the regulation of K^+^ and Cl^−^ channels (Garcia-Mata et al., 2003; Sokolovski et al., 2005), on the other hand it was suggested that NO can act as a negative regulator of ABA pathway via *S*-nitrosylation of the SnrK2,6/OST1 (Wang et al., 2015) and through the nitration of a Tyr residue of the PYR/PYL/RCAR ABA receptor complex (Castillo et al., 2015).

## H_2_S physiology in guard cells

This first report of the participation of H_2_S in guard cell signaling appeared in 2010, and since then several works have shown that this gasotransmitter induces stomatal closure in different plant species (García-Mata and Lamattina, 2010; Hu et al., 2014; Papanatsiou et al., 2015). In Arabidopsis, DES1 produces H_2_S in response to ABA. H_2_S, in turn, increases endogenous NO production (Scuffi et al., 2014). H_2_S-dependent stomatal closure is impaired in the *nia1/nia2* double mutant. Moreover, the expression of both genes was reported to be upregulated by H_2_S donors, suggesting that NR is involved in H_2_S-dependent NO production (Scuffi et al., 2014). However, Lisjak et al. (2010, 2011) showed that exogenous addition of H_2_S decreased ABA-dependent NO production and thus induced stomatal opening. Interestingly, it was reported that H_2_S also modulates Eth-dependent stomatal closure, but in this particular case, NO was reported to act upstream of H_2_S (Liu et al., 2011; Hou et al., 2013). There are other components, beside NR, that were pointed as targets of H_2_S during stomatal closure induction, among them: (i) the member of the multidrug resistance protein family AtMRP5, which was proposed as a modulator of Ca^2+^ and anion channels (Suh et al., 2007; García-Mata and Lamattina, 2010); (ii) 8-nitro cGMP, which reacts with H_2_S to form 8-mercapto cGMP to modulate [Ca^2+^]_cyt_ (Honda et al., 2015); and (iii) K${}_{\text{in}}^{+}$ channels, which are inactivated by H_2_S in an ABA-independent manner (Papanatsiou et al., 2015).

## The interplay between NO and H_2_S

The gasotransmitters NO and H_2_S not only share physicochemical similarities but they can also interact with each other in different biological systems and physiological conditions. Even though, there is still much to learn about: (i) the chemical nature of these interactions, (ii) the different products that can be potentially formed *in vivo* from the interaction, and (iii) the different biological outcomes. The study of the interaction between different gasotransmitters has kept the attention of researchers from different fields. As a result, different kinds of interactions have been described. There are interactions in which different gasotransmitters can act on the same molecular targets but having either the same or sometimes opposite outcomes (Mustafa et al., 2009b). An example of this in plants is the case of ABA-dependent induction of stomatal closure, where it has been reported that, on the one hand, ABA induces H_2_S production which in turn increases endogenous NO levels triggering stomatal closure (García-Mata and Lamattina, 2010; Scuffi et al., 2014), while on the other hand, it is reported that exogenous addition of H_2_S decreases ABA-dependent NO production, thereby producing the opening of the stomatal pore (Lisjak et al., 2010, 2011). There are other crosstalks in which different gasotransmitters produce the same outcome but acting on different molecular targets (Coletta et al., 2012). Such is the case of the regulation of guard cell K^+^ channels, where both NO and H_2_S selectively inactivate K${}_{\text{in}}^{+}$, however NO does this through a response that involves the release of Ca^2+^ from intracellular stores (Garcia-Mata et al., 2003), while H_2_S inactivates K${}_{\text{in}}^{+}$, mostly in an ABA and Ca^2+^ independent manner (Papanatsiou et al., 2015).

H_2_S and NO can also regulate each other source, by modulating the enzymatic production of the other. In animal systems H_2_S is able to down regulate NO production by inhibiting both constitutive and inducible NOS isoforms (Kubo et al., 2007) or to upregulate endothelial NOS (eNOS) dependent NO production (Predmore et al., 2011). In plants, it has been suggested that H_2_S induces NR-dependent NO production via the regulation of both *NIA1* and *NIA2* genes (data accessible at NCBI GEO database, accession GSE32566). The cross regulation of gasotransmitter sources has been also shown in the opposite sense. Zhao et al. (2001) have shown that NO donors upregulate the expression of the animal enzymatic H_2_S source, cystathionine-γ-lyase (CSE) and its consequent H_2_S production. In plants NO also upregulates H_2_S production during Eth-induced stomatal closure by increasing the expression of *AtL-CDes/AtD-CDes* genes (Liu et al., 2011, 2012).

The gasotransmitters can directly modify their specific targets by posttranslational modification (PTM) of the target protein. Nitric oxide can react with the thiol group of a cysteine residue to form S-nitrosocysteines residues (R-SNO) in a process known as S-nitrosylation, while H_2_S forms a persulfide group (R-SSH) in a process known as S-sulfhydration. There are proteins that can be modified by both gasotransmitters at the same cysteine residue. Interestingly, sulfhydration and nitrosylation can influence the protein function in different manners, S-nitrosylation modifications usually results in the inactivation of the protein while S-sulfhydration of the protein in many cases results in activating the biological function of the protein (Mustafa et al., 2009a; Jiang et al., 2010). Two examples of the different effects of the PTM in plants are the enzymes ascorbate peroxidase (APX) and glyceraldehydes-3P-dehydrogenase (GAPDH) whose activities are affected in different senses when they are S-nitrosylated or S-sulfhydrated, enabling enzymes play additional functions due to PTMs processes (Aroca et al., 2015).

The study of the interaction between H_2_S and NO is one of the current challenges for understanding the biology of these two gasotransmitters. The current knowledge shows that these two gases can interact at different stages of the signaling process, at different levels of their biosynthetic pathways and depending of the metabolic and redox status of the target cells (Cortese-Krott et al., 2014, 2015a; Lo Faro et al., 2014).

**Figure 1:**
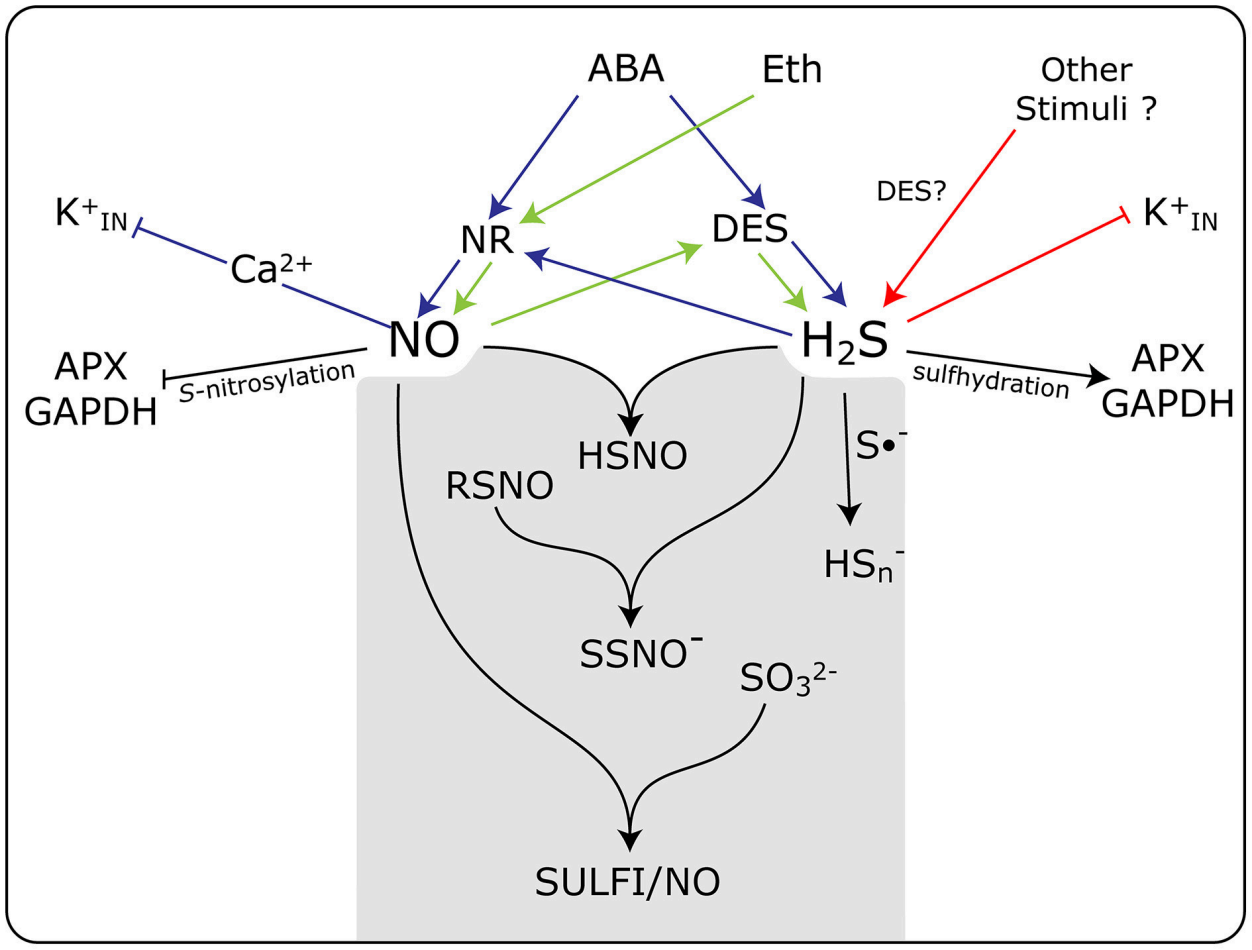
**Schematic model showing the action of NO and H_2_S and the interplay between them in guard cell signaling in response to different stimuli**. Both NO and H_2_S are produced enzymatically by nitrate reductase (NR) and L-cysteine desulfhydrase (DES), respectively, in response to abscisic acid (ABA), ethylene (Eth), and other stimuli. Some of the known targets of NO and H_2_S are inward rectifying K^+^ channels (K${}_{\text{in}}^{+}$), and the enzymes ascorbate peroxidase (APX) and glyceraldehyde-3-phosphate dehydrogenase (GAPDH). The gray box shows the chemical reactions between NO and H_2_S, and the formation of potential intermediates that could be involved in the regulation of guard cell physiology. HSNO, thionitrous acid; SSNO^−^, nitrosopersulfide; RSNO, nitrosothiols; Hs${}_{n}^{-}$, polysulfide; S•^−^, radical anion; SO${}_{3}^{2-}$, sulfite, and SULFI/NO, N-nitrosohydroxylamine-N-sulfonate. Blue arrows, ABA triggered events, green arrows Eth triggered events; red arrows participation of other stimuli. Arrow end, activation; blunt end, inactivation.

The existence of a direct chemical reaction between NO and H_2_S has gained strength in recent years. It is speculated that this interaction may result in the formation of some novel forms of nitrosothiols not yet fully characterized. In a recent paper, Cortese-Krott et al. (2015b) proposed that the interaction of NO and H_2_S would result in the formation of bioactive products at physiological pHs, emphasizing the formation of nitrosopersulfide (NO^−^), polysulfides (HS_n_), and N-nitroso-hydroxylamine-N-sulfonate (SULFI/NO). These compounds could regulate the bioavailability of NO and H_2_S by either the releasing or scavenging of each of them, which is depending on the relative concentrations of each one and the redox status of the cell (Cortese-Krott et al., 2015a) In a simplified schematic representation, Figure 1, we summarize the interplay and the close association existing between NO and H_2_S, and the formation of potential intermediates that could be involved in the regulation of guard cell physiology. These new discovered molecular forms might potentially explain the conflicting results concerning the roles of NO and H_2_S of influencing stomatal movement.

## Conclusions

The details of the biochemical interaction between NO and H_2_S are scarcely known in guard cells and in plant systems in general, in comparison to some physiological processes in animal systems (Kolluru et al., 2013; Lo Faro et al., 2014; Cortese-Krott et al., 2015a). However, the evidences presented in this study indicate that both gases can modulate stomatal movement acting independently, or in concerted action, in and ABA-triggered signaling cascade, or in ABA-independent manner. They can modulate the activity of the same molecular target by PTM of cysteine residues, and can even regulate the production and/or bioavailability of each other. In conclusion, it arises the point of the need to be cautious when drawing conclusions about the effects of either NO or H_2_S, unless the effect of both are studied together at the same biological conditions.

## Author contributions

Both DS and LL contributed in the writing of the manuscript, LL also contributed to give shape the idea of the opinion. CG made most of the writing and the figure and had the original idea.

## Funding

This work was financially supported through grants from the Universidad Nacional de Mar del Plata (UNMdP), PIP-240 from Consejo Nacional de Investigaciones Científicas y Técnicas (CONICET), and PICT-2013-3184 and PICT (CM) and PICT-2011-2383 (LL) from Agencia Nacional de Promoción Científica y Tecnológica (ANPCyT). CG and LL are researchers from CONICET.

### Conflict of interest statement

The authors declare that the research was conducted in the absence of any commercial or financial relationships that could be construed as a potential conflict of interest.
